# Exploring the potential of curry leaves on mercury‐induced hepatorenal toxicity in an animal model

**DOI:** 10.1002/fsn3.2683

**Published:** 2021-12-13

**Authors:** Muhammad Ijaz, Asma Arshad, Muhammad Ahmad Awan, Muhammad Rizwan Tariq, Shinawar Waseem Ali, Sajid Ali, Muhammad Shafiq, Saeed Ahmed, Muhammad Naveed Sheas, Madiha Iftikhar, Sheraz Ahmed, Muhammad Adnan Nasir, Ghazala Kausar, Ammad ul Islam Javed, Waseem Safdar

**Affiliations:** ^1^ National Institute of Food Science and Technology University of Agriculture Faisalabad Faisalabad Pakistan; ^2^ Quaid‐e‐Azam Medical College Bahawalpur Pakistan; ^3^ FMU Faisalabad Medical University Faisalabad Pakistan; ^4^ Department of Food Sciences University of the Punjab Quid‐i‐Azam Campus Lahore Pakistan; ^5^ Department of Biological Sciences National University of Medical Sciences Rawalpindi Pakistan; ^6^ Department of Diet & Nutritional Sciences The University of Lahore Islamabad Campus Islamabad Pakistan; ^7^ Department of Food Science Cholistan University of Veterinary and Animal Sciences Bahawalpur Pakistan; ^8^ University Institute of Diet & Nutritional Sciences The University of Lahore Gujrat Campus Gujrat Pakistan; ^9^ School of Food and Nutrition Minhaj University Lahore Lahore Pakistan

**Keywords:** curry, kidney, lipid profile, liver, toxicity

## Abstract

Herbal drugs play an imperative role in healthcare programs in developing countries. Curry leaves have wide medicinal importance and are used to treat various diseases traditionally. The current study was carried out to estimate the extent of mercury toxicity and the potential effect of curry leaves against defined toxicity. The study group comprised 24 rats weighing between 130 and150 g. Group 1 was kept normal, and group 2 was exposed to mercury at 0.4 mg/kg of body weight in the form of mercuric chloride (HgCl_2_). The group 3 animals were treated with curry leaves with a dosage of 300 mg/kg of body weight. Group 4 was treated with curry leaves along with mercury with a dosage of 300 and 0.4 mg/kg consecutively. After 28 days, the rats were killed. Blood sample of all groups were evaluated separately to determine the results of different parameters. The results show that ALP, AST, ALT, urea, bilirubin, and creatinine increased with mercury application and decreased with curry leaf exposure. SOD, CAT, GPx, and GR of the liver as well as the kidney depleted on mercury exposure whereas they increased with curry leaf application. HDL increased with curry leaf application and decreased with mercury treatment, while LDL, triglyceride, and cholesterol decreased with curry leaves and increased with mercury exposure. Organ index in mercury along with curry leaf application got close to normal.

## Graphical abstract

The extent of mercury toxicity and the potential effect of curry leaves were assessed against the defined toxicity. A total of 24 rats weighing between 130 and 150 g were exposed to mercury and treated with curry leaves for 28 days. Obtained data from this study showed that all the treatments have important effects on liver and kidney parameters, i.e., ALP, AST, ALT and urea, bilirubin, and creatinine increased with mercury application and decreased with curry leaf exposure. SOD, CAT, GPx, and GR of the liver as well as the kidney depleted on mercury exposure whereas increased with curry leaf application.

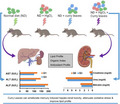

## 1 INTRODUCTION

Hepatorenal syndrome is a form of kidney dysfunction in individuals with progressive liver disease. Hepatorenal toxicity causes destruction of renal tissues due to cirrhosis. The primary cause of this toxicity is heavy metal overload, which enhances reactive oxygen species (ROS), which induces organ damage due to oxidative stress. Heavy metals react with cellular building blocks and damage them (Ghosh et al., 2012). Excessive consumption of mercury intensifies the formation of free radicals. The toxicity of mercury is attributed to its affinity with thiol‐containing molecules. This binding then results in the incidence of kidney and liver disorders. ROS and thiol‐containing molecules cause mercury toxicity that can be inhibited by antioxidants (Gado & Aldahmash, 2013). The main affected organs are the lungs, brain, gastrointestinal (GI) tract, and kidneys. Within a few hours, patients may suffer from dyspnea, cough, chills, chest tightness, fever, laziness, and GI problems. In humans and other mammals, the primary target is the kidneys where mercuric ions accumulate (Zahir et al., 2005; Zalups, 2000).

Heavy metals can injure cells, tissues, and organs. Even the least concentration of heavy metals can cause toxicity in food chains (Stummann et al., 2008). Industries, including petrochemicals, metallurgic processes, galvanic processes, paints foundry, ceramic industries, and plastic materials, are the main source of heavy metals (Zhang et al., 2016). Increased levels of mercury in water have adverse biological and pathological effects on marine creatures as determined by histopathological studies of the liver and kidney. The liver, brain, and kidneys are the organs most affected by mercury‐induced toxicity (Flora et al., 2008).

Herbal remedies showed an imperative role in healthcare programs in developing countries (Shankar & Ved, 2003). Curry leaves (*Murraya koenigii*) belonging to the family Rutaceae are used as a spice due to their distinctive flavor and aroma. Since ancient times, Indians have been using curry leaves (also called curry pata) as a spice. Found in Tarai areas and all over South India, the curry tree is also cultivated in Australia, Sri Lanka, Pacific Island, and China (Kesari et al., 2007). Phytochemical investigations revealed that curry leaves contain volatile oil, xanthotoxin, alkaloid, sesquiterpene, and glycozoline. The leaves of *M*. *koenigii* have incredible therapeutic value and are also used externally to cure eruptions (Handral et al., 2010; Pande et al., 2009). The leaves possess antidiabetic, hypoglycemic, antimicrobial, antioxidant, antibacterial, and anticarcinogenic properties (Ningappa et al., 2010).

Mercury is commonly used in barometers and thermometers (Emsley, 2011). Inorganic mercury (Hg) exists in the form of salts, mostly in mono and divalent forms (Johnson, 2004). High levels of mercury in water can negatively affect the pathology and biology of life forms (Adams et al., 2010). Traditionally, curry leaf decoction has been used as an antitumor, anti‐inflammatory, and antioxidative due to the high content of carbazole alkaloids. Its extensive applications in food industry as flavoring agent and in the pharma industry due to the presence of koenine, scopotin, calcium, isomahanine, thiamine, phosphorus, riboflavin, bismahanine, vitamin C, oxalic acid, carotene, and O‐methyl murrayamine increase its potential value in the global market (Dineshkumar et al., 2010). Moreover, curry leaves express antioxidative (Shukla et al., 2009), antitumor (Ito et al., 2006), anti‐inflammatory (Muthumani et al., 2009), hypoglycemic (Tembhurne & Sakarkar, 2009), antihyperglycemic, and hypolipidemic (Dineshkumar et al., 2010) effects. Carbazole alkaloids (Ito et al., 2006; Mishra et al., 2009) are considered to be the principal component of curry leaves. The leaves were used in traditional Indian medicine to cure various ailments (Chevallier, 1996; Sivarajan and Balachandran, 1994). Curry leaves can extend the shelf life of biscuits, which are made by a blend of wheat and sorghum flour (Emmanuel et al., 2012). The curry leaf powder is used as a spicy mixture in cooked rice, chapatti, and seasoned potatoes (Shanthala & Prakash, 2005). Keeping in view these benefits of curry leaves, this study was designed to determine the effective dosage of curry leaves for the reduction of kidney and liver toxicity.

## 2 MATERIALS AND METHODS

### 2.1 Sample preparation

Mature fresh curry leaves were purchased from the local market. The leaves were washed with tap water and shade‐dried at 28–30°C for 5–7 days. Thereafter, the leaf sample was ground to powder form using an electrical grinder.

### 2.2 Curry leaf extraction

About 500 g of the leaf powder was macerated with 1.5 L of methanol sequentially for 36 h. The sample was filtered using muslin cloth and the extract was separated from the leaf powder residue.

### 2.3 Solvent evaporation of extract

The methanolic extract was kept in a rotary vacuum evaporator at 40°C until complete evaporation of ethanol. After evaporation, the pure extract was stored in a refrigerator until further use.

### 2.4 Efficacy plan

**FIGURE 1:**
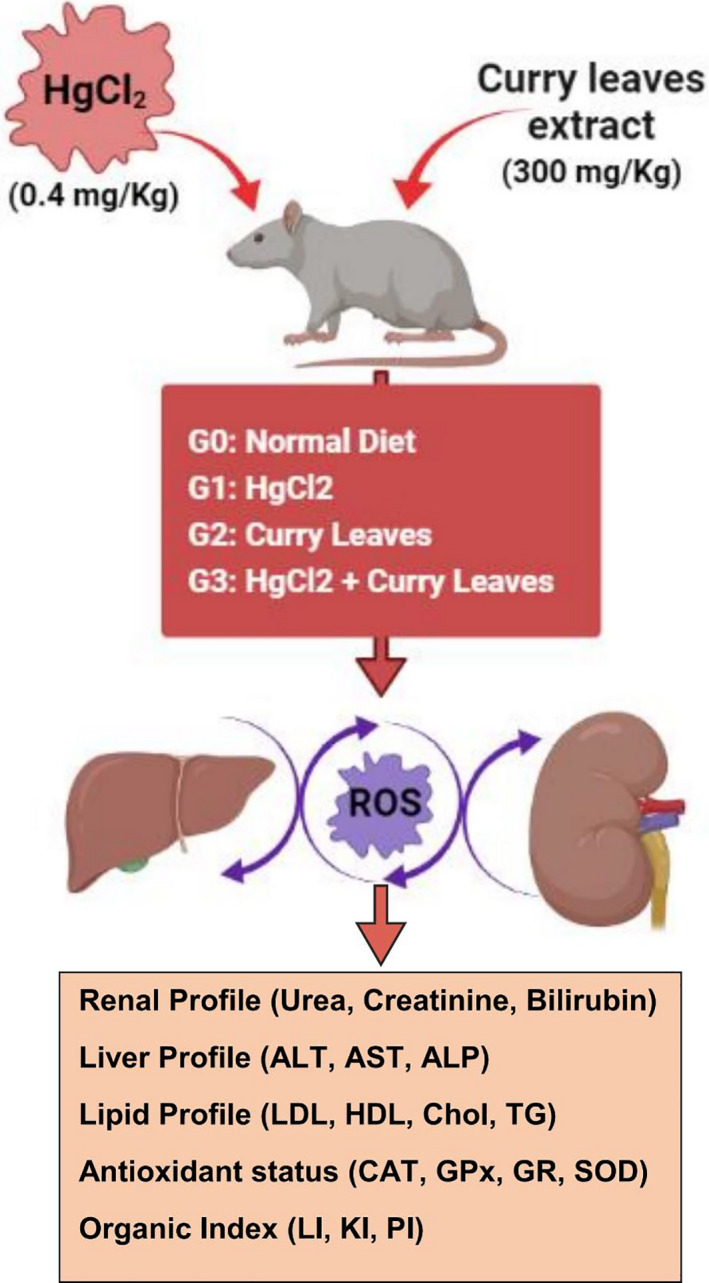
Schematic representation of methodology used to explore the effect of curry leaves on mercury‐induced hepatorenal toxicity. ALP, alkaline phosphatase; ALT, alanine aminotransferase; AST, aspartate aminotransferase; CAT, catalase; Chol, cholesterol; GPx, glutathione peroxidase; GR, glutathione reductase; HDL, high‐density lipoprotein; HgCl_2_, mercuric chloride; KI, kidney index; LDL, low‐density lipoprotein; LI, liver index; PI, pancreas index; SOD, superoxide dismutase; TG, triglycerides

Ethics approval was obtained from the Ethical Review Committee (ERC) of the University of Agriculture Faisalabad, Pakistan, before the start of the experiments. Male albino rats (*n* = 200; 130–150 g) purchased from the University of Agriculture Faisalabad were kept in cages. After adaptation, the rats were divided into four groups with each group consisting of 50 rats (Table 1). Figure 1 is the schematic representation of the efficacy plan. Rats in the first group (G_0_) served as the normal group and were fed a normal diet with distilled water. Rats in the second group (G_1_) were given mercuric chloride (HgCl_2_) orally at a dosage of 0.4 mg/kg of body weight and with normal daily feed. Rats in the third group (G_2_) were given an ethanolic extract of curry leaves orally at a dosage of 300 mg/kg of body weight along with 1% of sodium carboxymethyl cellulose and daily normal food. Curry leaves are hydrophobic in nature and hence sodium carboxymethyl cellulose was included to increase the bioavailability of curry leaves.

**TABLE 1:** Treatment plan

Treatments	Description
G_0_	Normal feed + distilled water
G1	Normal feed + distilled water + HgCl_2_ (0.4 mg/kg body weight)
G_2_	Normal feed + distilled water + extract of curry leaves (*Murraya koenigii*) (300 mg/kg of body weight) + 1% sodium carboxymethyl cellulose
G_3_	Normal feed + distilled water + mercuric chloride dose (0.4 mg/kg of body weight) + extract of curry leaves (*Murraya koenigii*) (300 mg/kg of body weight) + 1% sodium carboxymethyl cellulose

Rats in group 4 (G_3_) were exposed to a mixture of mercuric chloride (0.4 mg/kg of body weight), curry leaf extract (300 mg/kg of body weight), and 1% sodium carboxymethyl cellulose, along with the daily normal diet. After the 28‐day study period, rats were killed and blood samples were collected in sterilized vials.

### 2.5 Biochemical tests

#### 2.5.1 Liver and kidney function tests

Levels of alanine aminotransferase (ALT) and aspartate aminotransferase (AST), alkaline phosphatase (ALP), serum bilirubin, serum urea, and serum creatinine were calculated according to the methods of Reitman and Frankel (1957), Belfield and Goldberg (1971), Garber (1981), Wybenga et al. (1971), and Chromý et al. (2008) respectively.

### 2.6 Liver and kidney antioxidant status

The status of superoxide dismutase (SOD), catalase (CAT), glutathione peroxidase (GPx), and glutathione reductase (GR) of the kidney and liver was determined following the methods of Nishikimi et al. (1972), Abe et al. (1984), Paglia and Valentine (1967), and Factor et al. (1998) respectively.

### 2.7 Lipid profile tests

Serum lipid profile of rats like low‐density lipoprotein (LDL), high‐density lipoprotein (HDL), and serum total cholesterol was determined in accordance with Kim et al. (2011).

### 2.8 Organ index

Weight of the kidneys and pancreas was determined by a previously described method (Dyer et al., 2008). Weight of the liver was measured using the formula designed by Yang et al. (2005).

### 2.9 Statistical analysis

Mean values were calculated for each group. Data were analyzed with the Statistical Package for the Social Sciences (SPSS, version 25). One‐way ANOVA followed by the Dunnett's multiple comparison test was used for statistical analysis. *p* < .05 was considered statistically significant.

## 3 RESULTS AND DISCUSSION

The statistical analysis showed that the treatment effect (normal, mercury, curry leaves, and mixture) was highly significant on ALP (Table 2). The normal mean value of ALP was 129.7 IU/L that is increased with mercury (197.4 IU/L). The mean value of ALP becomes near normal with the application of curry leaves (136.5 IU/L), and with the application of mixture (mercury + curry leaves), the ALP value was 176 IU/L. The results indicate that the treatment effect (normal, mercury, curry leaves, and mixture) was highly significant on ALT. The level of ALT in the control group was 76.8 IU/L, which increased with mercury (145.6 IU/L). Curry leaves showed the best results, as the ALT levels in rats receiving curry leaves were near normal (74.3 IU/L); for the application of mixture (mercury + curry leaves), the ALT level was 111.9 IU/L. The treatment effect (normal, mercury, curry leaves, and mixture) was highly significant on AST. The normal value of AST is 79.9 IU/L, which was enhanced by the application of mercury (121.7 IU/L). The best result was obtained with the application of curry leaves (74.1 IU/L), which is near about to the normal value. With the application of mixture (mercury + curry leaves), the AST level was 99.5 IU/L.

**TABLE 2:** Liver function tests to explore the effect of curry leaves on mercury‐induced hepatorenal toxicity

Treatments	ALP (IU/L)	ALT (IU/L)	AST (IU/L)
G_0_	129.7 ± 1.42^d^	76.8 ± 0.64 ^c^	79.9 ± 0.92^c^
G_1_	197.4 ± 1.69^a^	145.6 ± 0.59^a^	121.7 ± 1.31^a^
G_2_	136.5 ± 1.08^c^	74.3 ± 0.21^c^	74.1 ± 0.84^d^
G_3_	176.0 ± 2.15^b^	111.9 ± 1.17^b^	99.5 ± 0.43^b^

Note

Means with different superscript letters are not significant at the 0.05% level of probability.

In the case of total bilirubin, the effect of treatment (normal, mercury, curry leaves, and mixture) was highly significant (Table 3). The normal value of bilirubin was 2.7 mg/dl. The value of bilirubin becomes near normal with the application of curry leaves (2.7 mg/dl) and with the application of mixture (mercury + curry leaves), the bilirubin value was 2.9 mg/dl. The level of bilirubin was high in rats receiving mercury (4.6 mg/dl). The effect of treatment (normal, mercury, curry leaves, and mixture) was also highly significant for urea. The normal value of urea was 6.1 mg/dl. The normal value increased with the application of mercury (7.6 mg/dl). The value of urea became near normal with the application of curry leaves (6.0 mg/dl), and with the application of mixture (mercury + curry leaves), the value of urea was 6.5 mg/dl. For creatinine, the treatment (normal, mercury, curry leaves, and mixture) had a highly significant effect. The value of the control group was 0.5 mg/dl. The value of creatinine in the group receiving curry leaves was 0.6 mg/dl. Application of mercury increased the level of creatinine (0.7 mg/dl) as did the application of mixture (mercury + curry leaves; 0.7 mg/dl).

**TABLE 3:** Kidney function tests to explore the effect of curry leaves on mercury‐induced hepatorenal toxicity

Treatments	Bilirubin (mg/dl)	Urea (mg/dl)	Creatinine (mg/dl)
G_0_	2.7 ± 0.01^b^	6.1 ± 0.02^c^	0.5 ± 0.01^b^
G_1_	4.6 ± 0.01^a^	7.6 ± 0.03^a^	0.7 ± 0.00^a^
G_2_	2.7 ± 0.03^b^	6.0 ± 0.02^c^	0.6 ± 0.00^ab^
G_3_	2.9 ± 0.01^b^	6.5 ± 0.03^b^	0.7 ± 0.01^a^

Note

At the level of 0.05% probability, there is no single letter sharing by means.

Accumulation of mercury in the biological system causes a lot of variation that promotes antagonistic health effects in rats (Huang et al., 2008). Mercury‐treated rats show variation in morphological characteristics in the liver and kidneys, such as deterioration, hemo sinusoids, loss of hepatic tissue patterns along with hypercirculation in glomeruli, intratubular degeneration, and hemorrhage. The concentration of serum creatinine and urea helps in measuring the functionality of the kidney and the structural integrity of the renal tubules. An increase in the level of creatinine and urea in mercury‐treated rats indicated nephrotoxicity (Franciscato et al., 2011). Raised level of urea and creatinine damages renal tubules, which is clearly marked by degenerative changes in kidney tissues when compared with the normal group (Gado & Aldahmash, 2013; Glaser et al., 2010; Oriquat et al., 2012), an observation that also holds true for serum biochemical parameters and histopathological variations resulting from mercury‐induced nephrotoxicity.

Enhanced serum levels of ALP, AST, and ALT indicate possible mercury‐induced hepatotoxicity in rats. The increased levels of these enzymes are significant markers of hepatocellular destruction (Abdel‐Moneim et al., 2011). The level of liver enzymes is also increased Emamuzo et al., 2010). Sheikh et al. (2013) affirmed that inorganic mercury induces hepatotoxicity. However, curry leaves stopped mercury‐induced hepatic and renal damage as observed by the decreased levels of hepatic enzymes. Sindhu et al. (2012) found that the levels of enzymatic and non‐enzymatic antioxidants returned to near normal with oral administration of berberine to paracetamol‐treated rats.

In the case of LDL, the treatment effect (normal, mercury, curry leaves, and mixture) was highly significant (Table 4). The best result was obtained by the application of curry leaves (32.9 mg/dl) that was close to the value of the control group (45.1 mg/dl). The level of LDL increased by mercury application (66.9 mg/dl), and the level of LDL was 49.9 mg/dl with the application of mixture (mercury + curry leaves). Statistical analysis of cholesterol indicated that the treatment effect (normal, mercury, curry leaves, and mixture) was highly significant. The normal value of cholesterol was 84.1 mg/dl that increased with the application of mercury (106.52 mg/dl). The value of cholesterol was reduced by the application of curry leaves (61.1 mg/dl) and that was almost close to the value of the normal group. With the application of mixture (mercury + curry leaves), the cholesterol level was 81.5 mg/dl. In the case of HDL, results showed that the treatment effect (normal, mercury, curry leaves, and mixture) was highly significant. The normal level of HDL was 24.9 mg/dl. The normal level was decreased in mercury‐treated rats (22.8 mg/dl), but the level of HDL increased with the application of curry leaves (29.2 mg/dl). With the application of mixture (mercury + curry leaves), the level of HDL was 24.9 mg/dl. For triglyceride, the treatment effect (normal, mercury, curry leaves, and mixture) was highly significant. The normal value of triglyceride in the control group was 51.4 mg/dl, which was increased by the application of mercury (83.6 mg/dl). The level of triglyceride reduced in rats that were receiving curry leaves (44.5 mg/dl), a value close to the level of the control group. With the application of mixture (mercury + curry leaves), the level of triglyceride increased to 67.8 mg/dl.

**TABLE 4:** Lipid profile tests to explore the effect of curry leaves on mercury‐induced hepatorenal toxicity

Treatments	LDL (mg/dl)	Triglyceride (mg/dl)	Cholesterol (mg/dl)	HDL (mg/dl)
G_0_	45.1 ± 0.68^c^	51.4 ± 0.76^c^	84.1 ± 0.82^b^	24.9 ± 0.62^b^
G_1_	66.9 ± 0.91^a^	83.6 ± 1.21^a^	106.6 ± 1.67^a^	22.8 ± 0.27^c^
G_2_	32.9 ± 1.04^d^	44.5 ± 0.69^d^	61.1 ± 1.24^d^	29.3 ± 0.94^a^
G_3_	49.9 ± 0.51^b^	67.8 ± 0.66^b^	81.5 ± 1.04^c^	24.9 ± 0.59^b^

Note

At the level of 0.05% probability, there is no single letter sharing by means.

In this study, LDL and cholesterol levels decreased and HDL levels increased in rats treated with curry leaves. These results are in accordance with those of Jayaweera et al. (2018), who observed that orally administered curry leaves significantly decreased serum cholesterol levels. Akila et al. (1998) found that curry leaves increased HDL levels and lowered cholesterol, suggesting that curry leaves might mobilize extrahepatic cholesterol to the liver where its breakdown or elimination is completed. Arafa (2005) stated that curry leaf extracts lower cholesterol levels. The mechanism of lowering LDL and cholesterol levels was not well known in earlier research. One plausible reason is that curry leaves decrease serum cholesterol levels by inhibiting absorption of dietary cholesterol. Molly et al. (2017) proposed that curry leaves increased HDL and lowered cholesterol and LDL levels.

In the case of organ index, the statistical results of pancreas index indicated that the treatment effect (normal, mercury, curry leaves, and mixture) was highly significant (Table 5). The normal percentage of pancreas (0.019%) was increased in rats by mercury application (0.021%). Pancreas index was reduced in rats administered curry leaf extract (0.018%). The level of pancreas index was 0.020% with the application of mixture (mercury + curry leaves). The statistical results of liver index showed that the treatment effect (normal, mercury, curry leaves, and mixture) was highly significant. The normal percentage of liver index was 3.6%. The percentage of liver index was near normal with the application of curry leaves (3.6%), and with the application of mixture (mercury + curry leaves), the percentage of liver index was 3.7%. The highest percentage of liver index in mercury‐treated rats was 3.7%. In the organ index, the statistical results of kidney index indicated that the treatment effect (normal, mercury, curry leaves, and mixture) was highly significant. The normal percentage of kidney index was 0.03% in the control group that was enhanced by mercury application (0.03%). The percentage of kidney index decreased with the application of curry leaves (0.03%) that was close to the normal percentage, and with the application of mixture (mercury + curry leaves), the kidney index was 0.03%.

**TABLE 5:** Organ index to explore the effect of curry leaves on mercury‐induced hepatorenal toxicity

Treatments	Liver index (%)	Kidney index (%)	Pancreas index (%)
G_0_	3.6 ± 0.24^b^	0.032 ± 0.00^c^	0.018 ± 0.00^bc^
G_1_	3.7 ± 0.17^a^	0.033 ± 0.00^a^	0.021 ± 0.00^a^
G_2_	3.6 ± 0.16^c^	0.031 ± 0.00^c^	0.017 ± 0.00^c^
G_3_	3.7 ± 0.09^a^	0.032 ± 0.00^b^	0.020 ± 0.00^ab^

Note

At the level of 0.05% probability, there is no single letter sharing by means.

The antioxidant enzyme status of the liver was evaluated by measuring the status of antioxidant enzymes. The statistical analysis of SOD indicated that the treatment effect (normal, mercury, curry leaves, and mixture) was highly significant (Table 6). The SOD value increased with the application of curry leaf extract in rats (1653.1 U/mg) that was close to the normal value of SOD in the control group (1231.6 U/mg). The normal value of SOD reduced in mercury‐treated rats (1100.8 U/mg), and with the application of mixture (mercury + curry leaves), the value of SOD increased (1734.4 U/mg). In the case of catalase (CAT), the results indicated that the treatment effect (normal, mercury, curry leaves, and mixture) was highly significant. The normal level of CAT was 0.07 U/mg. The CAT level was decreased by the application of mercury (0.07 U/mg). The level of CAT became near normal with the application of curry leaves (0.09 U/mg), and with the application of mixture (mercury + curry leaves), the value of CAT was 0.08 U/mg. The results of glutathione peroxide (GPX) indicated that the effect of treatment (normal, mercury, curry leaves, and mixture) was highly significant. The normal GPx value (0.9 U/mg) was reduced to 0.7 U/mg with the application of mercury. The value of GPx increased to near normal with the application of curry leaves (0.9 U/mg). However, the value of GPx was 0.7 U/mg with the application of mixture (mercury + curry leaves). For GR, the statistical results indicated that the treatment effect (normal, mercury, curry leaves, and mixture) was highly significant. The normal value of GR is 7.01 µmol/mg that is readily reduced by mercury (5.79 µmol/mg). The value of GR increases with the application of curry leaves (8.71 µmol/mg) whereas it decreases with the application of mixture (mercury + curry leaves; 8.47 µmol/mg).

**TABLE 6:** Liver antioxidant status to explore the effect of curry leaves on mercury‐induced hepatorenal toxicity

Treatments	CAT (U/mg)	GPx (IU/L)	GR (µmol/mg)	SOD (U/mg)
G_0_	0.072 ± 0.00^bc^	0.86 ± 0.01^b^	7.0 ± 0.04^b^	1231.6 ± 23.8^c^
G_1_	0.065 ± 0.00^c^	0.65 ± 0.02^d^	5.8 ± 0.05^c^	1100.8 ± 18.5^d^
G_2_	0.085 ± 0.00^a^	0.99 ± 0.01^a^	8.7 ± 0.09^a^	1653.1 ± 38.2^b^
G_3_	0.080 ± 0.00^ab^	0.78 ± 0.01^c^	8.5 ± 0.12^a^	1734.4 ± 32.4^a^

Note

At the level of 0.05% probability, there is no single letter sharing by means.

As regards the antioxidant enzyme status of the kidney, treatment (normal, mercury, curry leaves, and mixture) had a highly significant effect on SOD (Table 7). The normal value of SOD was 1913.8 U/mg. The normal value decreased (1352.3 U/mg) in mercury‐treated rats. In the group receiving curry leaves + mercury, a maximum value of 1713.8 U/mg for SOD was obtained. While the level of SOD was 1711.8 U/mg with the application of mercury + curry leaves, the normal value of CAT was 0.097 U/mg. The value obtained by the application of curry leaves was 0.09 U/mg, which was near normal, and with the application of mixture (mercury + curry leaves), the value of CAT was 0.09 U/mg. The level of CAT was highest (0.06 U/mg) in mercury‐treated rats. The results of GPx enzyme in the kidney indicated that the treatment effect (normal, mercury, curry leaves, and mixture) was highly significant. GPx enzyme level increased with the curry leaf application in rats (0.71 U/mg), which was 0.64 U/mg, but with the application of mercury, it was decreased (0.56 U/mg), and with the application of mixture (mercury + curry leaves), the value of GPx was 0.81 U/mg. The statistical results showed that the treatment (normal, mercury, curry leaves, and mixture) had a highly significant effect on GR. The normal value of GR was 7.22 µmol/mg, which readily decreased with mercury (3.7 µmol/mg). The value of GR rose with the application of curry leaves (7.27 µmol/mg), and with the application of mixture (mercury + curry leaves), the value of GR was 6.15 µmol/mg.

**TABLE 7:** Kidney antioxidant status to explore the effect of curry leaves on mercury‐induced hepatorenal toxicity

Treatments	CAT (U/mg)	GPx (U/mg)	GR (µmol/mg)	SOD (U/mg)
G_0_	0.097 ± 0.00^a^	0.64 ± 0.01^c^	7.2 ± 0.08^a^	1913.8 ± 18.5^a^
G_1_	0.060 ± 0.00^b^	0.56 ± 0.00^d^	3.7 ± 0.04^c^	1352.3 ± 21.7^c^
G_2_	0.092 ± 0.00^a^	0.71 ± 0.01^b^	7.3 ± 0.09^a^	1711.8 ± 28.1^b^
G_3_	0.091 ± 0.00^a^	0.81 ± 0.00^a^	6.1 ± 0.01^b^	1713.8 ± 15.9^b^

Note

At the level of 0.05% probability, there is no single letter sharing by means.

**FIGURE 2:**
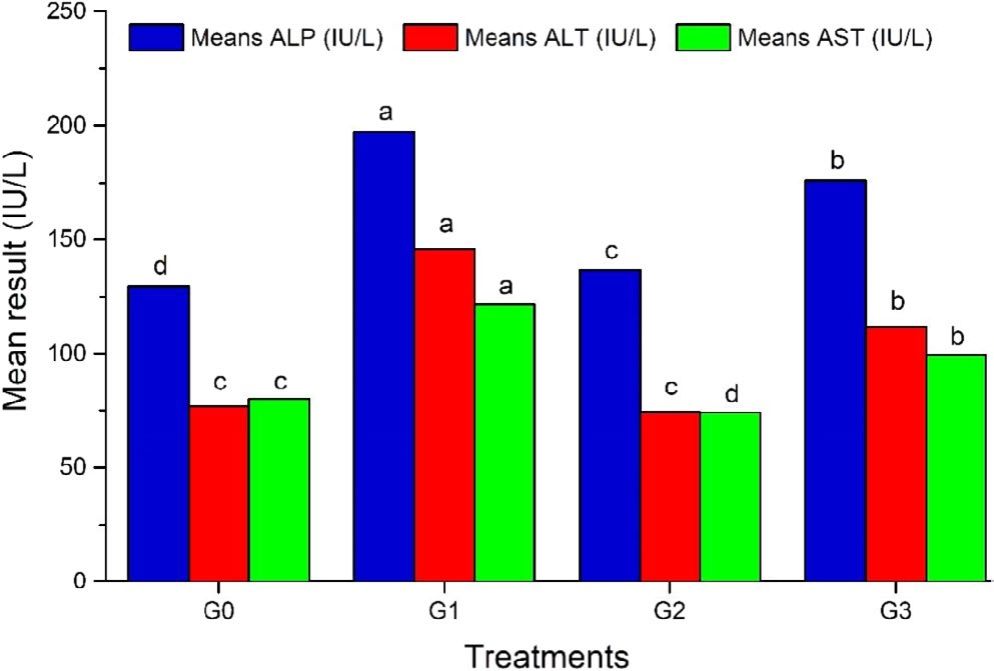
The overall mean effect of curry leaves on mercury‐induced hepatorenal toxicity. Results with different superscripts were significantly different from each other (*p > *.05). G0: control fed normal diet; G1: introduced 0.4 mg/kg body weight HgCl_2_; G2: introduced extract of 300 mg/kg body weight curry leaves; G3: mixture of HgCl_2_ (0.4 mg/kg) + extract of curry leaves (300 mg/kg) + 1% sodium carboxymethyl cellulose

The reducing power of curry leaves due to the presence of a cocktail of various phytochemicals and vitamins ultimately scavenges heavy metal toxicity. Increased ROS were reported in previous studies during Hg exposure. Subsequently, ROS attack almost all cell components including membrane lipids and produce lipid peroxidation (LPO). Flora et al. (2008) showed that mercury toxicity produces free radicals mainly in the kidney and liver tissues. They found that the unifying factor in determining toxicity and carcinogenicity for metals is the generation of reactive oxygen and nitrogen species. Mercury‐treated rats demonstrated many oxidative stress markers such as NO and LPO in the kidneys and liver. Therefore, it was suggested that oxidative stress is involved in organ dysfunction due to mercury toxicity. A previous study suggested that mercury exposure increased the level of ROS, which mostly affects cell components such as lipids (Tang et al., 2006). GPx, GR, SOD, and CAT are recognized as the first line of defense against free radical injury (Sumathi et al., 2017). The present study shows that in mercury‐treated rats, the level of antioxidant enzymes was significantly reduced when compared with the control group, which shows that mercury triggers oxidative stress (Figure 2). SOD and GPx expression was downregulated on mercury exposure, suggesting that the accumulation of mercury in biological systems reduced the level of antioxidants.

## 4 CONCLUSION

After entering the body, heavy metals can penetrate into tissues and cause oxidative damage through ROS. Certain health hazards and pathological conditions arise owing to the dysfunction of biochemical and physiological processes. Exposure to heavy metals such as mercury caused degenerative changes in liver enzymes (ALT, AST, and ALP) and renal biomarkers (urea, bilirubin, and creatinine). Most of the conventional synthetic drugs have adverse side effects; herbal remedies can be safer. Curry leaf extract significantly normalized liver and kidney parameters in the animal model exposed to mercury. The antioxidant level in the kidney and liver was depleted in the group exposed to mercury, whereas antioxidants such as CAT, GR, GPx, and SOD significantly improved in the group exposed to mercury along with curry leaf extract. The result revealed a significant increase in HDL in curry leaf‐treated rats while exhibiting lower levels of triglycerides, LDL, and total cholesterol when compared with the control group. These results suggest that curry leaves are an integral part of food grade remedies for treating, curing, or preventing occupational health hazards caused by heavy metal exposure.

## CONFLICT OF INTEREST

The authors do not have any conflict of interest.

## ETHICAL APPROVAL

The protocol and procedures employed were ethically reviewed and approved by the Ethical Review Committee (ERC) of the University of Agriculture Faisalabad, Pakistan (letter number: ERC/2020‐069), Quaid‐e‐Azam Medical College Bahawalpur, Pakistan (QMC/BWP/2020‐AH‐20210524), and Department of Food Sciences, University of the Punjab, Quid‐i‐Azam Campus, Lahore, Pakistan (PU‐20J14214).

## Data Availability

The data that support the finding of this study are available from the corresponding author upon reasonable request.
